# A review of plastic waste nanocomposites: assessment of features and applications

**DOI:** 10.1186/s11671-024-04062-0

**Published:** 2024-07-06

**Authors:** Ida Rasilainen, Ville Lahtela, Timo Kärki

**Affiliations:** 1 Fiber Composite Laboratory, LUT School of Energy Systems, Lappeenranta-Lahti University of Technology LUT, Yliopistonkatu 34, 53851 Lappeenranta, Finland; 2 SCI-MAT Research Platform & Fiber Composite Laboratory, LUT School of Energy Systems, Lappeenranta-Lahti University of Technology LUT, Yliopistonkatu 34, 53851 Lappeenranta, Finland

**Keywords:** Polymer nanocomposite, Nanocomposite, Plastic waste, Nanomaterial, Recycling

## Abstract

Hundreds of millions of metric tons of plastic waste are generated globally every year. Processing waste into secondary raw material is preferred over energy production and landfilling. However, mechanical recycling generally deteriorates the properties of plastic waste limiting its range of potential applications. Nanocomposite fabrication is a solution to recycle plastic waste into value-added applications due to improved properties generated by nanomaterial reinforcement, however received little study. The aim of this review is to present the current status, identify research gaps and provide topics for further research of polymer nanocomposites prepared from plastic waste in respect to utilized materials, processing methods, enhanced properties, sustainability, economics, nanomaterial safety, and applications. It is found that morphological, mechanical, thermal, flame retardancy, physical, barrier, electrical and shielding properties of plastic waste can be enhanced with low loadings of different nanomaterials making them promising materials for various applications including electronic, shielding, thermal, packaging, filtration, and water treatment. Utilization of plastic waste instead of virgin polymers can be beneficial in respect to economics and sustainability, but the energy intensive and expensive production of the most nanomaterials, and the plastic waste pretreatment methods can negate these benefits. To enhance sustainability, further research should be conducted on utilization of energy friendly nanomaterials in plastic waste nanocomposites. Further research is needed also on polymer nanocomposite safety because of the unknow composition of the plastic waste and the potential for nanomaterial release during nanocomposite’s life cycle. All in all, further research and national regulations and guidance are needed on virgin polymer and plastic waste nanocomposites.

## Introduction

Plastics are a rapidly expanding field, and various types of plastic material are used in many different applications due to the material’s low density, good strength-to-weight ratio, thermal and electrical insulation properties, durability and moldability, among others [1]. However, generated amount of plastic waste is currently around 350 million metric tons worldwide annually while increasing every year indicating huge problems with the waste stream as only 9% is recycled, whereas 12% is incinerated, and the major share is landfilled, leaching out toxic chemicals into the environment [1, 2]. As determined in the waste hierarchy, which is the foundation for the European Union’s (EU) waste management strategy, recycling waste into secondary raw materials is favored over utilization as energy and landfilling [3].

Mechanical recycling is the most common solid plastic waste recovery process that involves various steps such as collection, separation, cleaning, drying, shredding and extrusion [1, 4, 5]. It is considered the simplest way to provide second life for the plastic waste products [6] while being energy-effective [1]. However, the melt processing might deteriorate the properties of the plastics hence limiting their further use in advanced applications [1, 6]. Mechanical recycling of plastic waste exhibit unsatisfactory properties mainly because of the macromolecules suffers from degradation due to melt processing, and because of the variating quality of plastic waste stream [6]. Mechanical treatment of plastic waste, even after careful separation and purification, produces blends with mixture of unknow ratio of different polymers, which are usually immiscible and incompatible with each other. Another plastic waste recycling method is chemical recycling, which can be classified into gasification, pyrolysis, fluid-catalysed cracking and hydrocraking [5]. In chemical recycling, monomers are recovered from plastic waste and further utilized to prepare new plastic products [5, 7]. Advantage of plastic waste recycling using chemical recycling is the high-quality material obtained that can be used in value-added applications. On the other hand, chemically recycled polymers are expensive compared to virgin ones, hindering their benefits [5].

One possible solution for plastic waste management is to utilize recycled polymers in value-added products using nanocomposite fabrication, thus generating material that is qualitative more valuable than the waste material [1, 8]. Polymer matrices reinforced with nanomaterials, which are less than 100 nm in size in at least in one dimension, are defined as polymer nanocomposites (PNC) [9]. In this review, PNCs in which plastic waste is used as a matrix are termed plastic waste nanocomposites (plastic waste NC).

Low loadings of nanomaterials in waste-derived or virgin polymers, in general from 0.1 to 5 wt%, have been found to improve mechanical, thermal, electrical, physical, rheological, optical, ablation, flammability, permeability, charge dissipation, magnetic, catalytic, and other properties due to nanomaterials’ large surface-to-volume area [10–12]. The use of nanomaterials in plastic waste not only improves several material properties, but the nanomaterial can also act as a blend compatibilizer while offering light weighted structure [11, 13]. However, the quality, possible applications, and cost of PNCs are greatly influenced by the type of plastic waste and nanomaterial, the nanomaterial loading, and the processing method utilized [1]. Moreover, in order to obtain superior properties, the nanomaterials should be distributed uniformly to polymer matrix without agglomeration [9], which is one of the main challenges facing PNC processing [12].

Other challenges facing utilization, production and properties of PNCs include defining suitable polymer-nanomaterial combinations, uneven quality of the plastic waste including unknow impurities, ensuring the cost-effectiveness of PNCs at industrial scale as mass production of nanomaterials is complex and expensive, and evaluating their safety for human health and the environment as the nanoscale particle size allows nanomaterials to diffuse into biological systems, which is not possible with larger particle sizes [12]. Generally, nanomaterials are considered safe when encapsulated in a polymer matrix, and, moreover, the definition of “nanomaterial” only defines material’s particle size, and not all nanomaterials are toxic [14]. Nevertheless, nanomaterial release through PNC’s lifecycle is possible and yet very little studied including lack of national regulations and guidance [15].

In a literature search for this review conducted in the Scopus database in 2023 using “polymer nanocomposite” as a search term for the article title, abstract and keywords, around ten thousand research papers were found. It was clearly seen that the number of publications per year increased linearly post-2007, and in 2023, over 800 PNC-related documents were published. In contrast, in a search conducted in 2023, only around 150 publications related to plastic waste nanocomposites were found when the following search criteria were utilized: ("recycled polymer" OR "recycled plastic" OR "waste plastic" OR "plastic waste" AND nanocomposite). In addition, only around 50 documents out of the 150 were related to polymer nanocomposites with plastic waste as the main material in the polymer matrix. Only thermoplastic matrices were noted. Many of the studies, which were not suitable for this review, dealt with either biodegradable polymers or considered the issue of plastic waste being converted into carbon-based nanomaterials.

Additional searches were carried out in the Scopus database in 2023 related to safety, risks, toxicity, and regulations of polymer nanocomposites for the article title, abstract and keywords, with search terms of (”polymer nanocomposite” OR PNC AND safety), (”polymer nanocomposite” OR PNC AND risk), (plastic OR polymer AND nanocomposite AND product AND safety), (plastic OR polymer AND nanocomposite AND harm OR toxi*), and (plastic OR polymer AND nanocomposite AND legislation OR regulation). A total of 2700 results were found from the searches. Each search was sorted by “relevance” out of which the suitability for the review were evaluated based on the content in the title and/or abstract of the first hundred appeared papers. Finally, around twenty most suitable research papers were selected for the review. It must be highlighted that most of the papers dealing with the safety aspects were related to virgin polymer nanocomposites rather than plastic waste nanocomposites.

This literature review presents the current status of research on polymer nanocomposites prepared from plastic waste with inclusion of different nanomaterials and is conducted based on examination of around 70 published research papers. The goal of the work is to provide comprehensive information about materials, plastic waste preparation procedures, polymer nanocomposite processing methods, studied material properties, applications, and the sustainability, economics, and safety of plastic waste nanocomposites. Research gaps and further research topics are also identified.

The paper is organized such that common materials used in plastic waste nanocomposites are first introduced, followed by the descriptions of the main processing methods, including plastic waste preparation, nanomaterial dispersion methods, and secondary manufacturing steps. Material properties are divided into morphological, mechanical, thermal, physical, and electrical properties. Additionally, applications, sustainability, safety, and economic aspects of plastic waste nanocomposites are presented. The paper concludes by presenting the current state of PNCs including suggestions for further research.

## Materials for plastic waste nanocomposites

### Plastic waste

Solid plastic waste can be divided into post-consumer waste and post-industrial waste, which of the latter one is typically leftover materials from production being often clean and considered as a high-quality plastic waste grade [5]. Post-consumer plastic waste, on the other hand, consist of plastic products that are discarded by consumers [5]. Over half of the plastic items produced worldwide are used in the packaging and construction sector, with shares of 44% and 18%, respectively [16]. The automotive industry, electronics, household, and leisure and sport sectors utilize huge amounts of plastics as well [16]. The most produced plastic grades globally in 2021 were polypropylene (PP), low-density polyethylene (LDPE), polyvinyl chloride (PVC), high-density polyethylene (HDPE), polyethylene terephthalate (PET), polyurethane (PU), and polystyrene (PS), with shares of 19.3%, 14.4%, 12.9%, 12.5%, 6.2%, 5.5%, and 5.3%, respectively [16]. The packaging sector utilizes mostly LDPE, HDPE, PP, and PET, while PVC is the major plastic grade in the construction and building industry according to Plastics Europe [16]. These plastic grades are typically disposed into landfills, incinerated due to their non-biodegradable nature, or recycled into secondary materials [1]. Based on data acquired in this review, HDPE [8, 10, 13, 17–24], PET [25–34], PS [24, 35–38], PP [39–42] and PVC [35, 36, 43, 44] have been the most studied plastic waste grades combined with different nanomaterials to prepare plastic waste nanocomposites. Other studied plastic waste grades reinforced with nanomaterials have been expanded polystyrene (EPS) [45, 46], polycarbonate (PC) [47], vulcanized nitrile butadiene rubber [44] and nylon 6,6 [48]. In the study of Xie et al. [49], aluminum-plastic packaging waste was used as a matrix in plastic waste NC.

The main plastic waste sources for PNC processing considered in the current work have been different post-consumer items, including bottles and containers, bags, packaging, and other plastic products, which have been purified before nanocomposite processing. Also, recycled polymers have been used as a source of plastic waste [8, 19, 27, 29, 33], indicating the quality to be close to high-quality industrial plastic waste rather than post-consumer plastic waste. Bottle waste have been source for recycled HDPE [21], PET [25, 26, 30–32] and PP [40], plastic bags and films for HDPE [13, 21, 22], packaging for PP [42], PS [37, 38], HDPE [13, 18], and EPS [45], cable tie waste for nylon 6,6 [48], and other plastic products for HDPE [20]. However, in most of the above-mentioned studies, the plastic waste is carefully selected, sorted, and purified before nanocomposite processing, characterizing the condition of post-industrial waste rather than post-consumer plastic waste. Typically, post-industrial plastic waste is clean, and the composition of the polymers is known, whereas the post-consumer plastic waste consists of unknown composition of mixed plastic waste which might be also contaminated with other materials such as food and metals, among others [5]. The separation of plastic waste is challenging because some polymer grades, such as PET and PVC, are difficult to identify from each other [11, 50]. The presence of several polymer grades in a product provides also a challenge due to their different melting temperatures, leading to poor compatibility and mechanical properties [13].

Some of the studies considered in this review prepared a polymer blend matrix from recycled HDPE combined with PET (25 wt%) [8, 17], PP (12–15 wt%) [13, 18], or ethylene–vinyl acetate (EVA) (30 wt%) [19], and from recycled PET combined with PLA waste [26], which reflect better the realistic composition of plastic waste compared to the publications where purified single polymers were used as a plastic waste source. In addition, Velásquez et al. [25] and Velásquez et al. [39] mixed recycled polymers, PET and PP, respectively, with their virgin polymers to compensate the deteriorated material properties of recycled polymers caused by recycling practice before nanocomposite processing with the nanoclay.

### Nanomaterials with plastic waste

Nanomaterials, also referred to nanofillers, have unique properties that differ from their larger particle size equivalents due to their large surface area per volume allowing them to make numerous bonds with surrounding molecules compared to micron scale particles [12]. Due to these multiple bonds, extremely solid molecular structures can be created improving the mechanical properties of the composite material [12].

Nanomaterials can be classified based on their geometry into particles, tubes, layered structures, and arrays, where the number of dimensions at nanoscale are 0, 1, 2, and 3, respectively. Types of nanomaterials used with polymers include carbon-based materials, for example, carbon black, fullerene, carbon nanotubes (CNTs), graphene and graphite, metallic particles, such as silver, copper, and aluminum, clay-based nanomaterials, referred to as nanoclays, for example, montmorillonite, halloysite, sepiolite and laponite, and natural nanomaterials, such as nanocellulose [51]. Carbon-based nanomaterials are used to enhance the electrical, thermal, and mechanical properties of the polymers [52]. Titanium dioxide and zinc oxide are used to improve optical and strength properties, whereas nitrides and carbides enhance hardness and wear resistance of the polymer matrix. Nanoclays enhance flame resistance, thermal stability and mechanical properties of the polymer, and nanometals are used for electric and thermal conductivity enhancement [12, 51].

Nanomaterial distribution into the polymer matrix can be divided into layered and dispersed type according to the nature of the nanomaterial’s structures. A special feature of layered nanoclays is that their layered structure allows the polymer matrix to pass through the clay galleries, which is called intercalation, or to distribute the stacked structure of the clay, which is known as exfoliation [51]. In order to achieve exfoliation and proper dispersion into the polymer matrix, nanoclays require modification to make their surface more organophilic [53].

According to López de Dicastillo et al. [54], nanoclays are the most used nanomaterials for post-consumer plastic waste reinforcement but also graphene and carbon nanotubes (CNTs) have been utilized. A similar observation was made in the current work as the most common nanomaterials used with plastic waste have been nanoclays [8, 13, 17–19, 24–26, 39, 42] and carbon-based nanomaterials [8, 10, 23, 29–31, 33, 36, 37, 44] with the focus on multi-walled carbon nanotubes (MWCNTs). Interestingly, none of the reviewed studies considered carbon black as a nanomaterial to improve the properties of plastic waste, even though it is widely used with virgin polymers. Based on the literature search conducted for this work, among nanoclays and CNTs, also nanosilica [41], cellulose nanocrystal [27], aluminum oxide nanoparticle (NP) [20], magnesium hydroxide NP [34], copper [28] and copper oxide NPs [21, 22], bismuth oxide NP [43], titanium dioxide NP [45, 46], and nickel zinc (NiZn) ferrite NP [35] have been combined with plastic waste to produce plastic waste nanocomposites. In addition, Ismail et al. [40] reinforced PP waste with ball milled coconut shell waste.

### Compatibilizers with plastic waste nanocomposites

Compatibilizers are used with the PNCs to enhance the compatibility of the polymer matrix and the nanomaterials because incompatibility of a hydrophobic matrix and hydrophilic nanomaterials will weaken nanomaterial dispersion and hence prevent improvement in the properties of the composite [17]. Therefore, good interaction between the matrix and the nanomaterials is important in order to achieve the desired mechanical properties [17, 27]. When using nanoclay, which is a common nanomaterial used with PNCs, its surface is typically chemically modified with organic surfactants to allow polymer intercalation between the clay platelets, thus enhancing their compatibility and improvement in properties [25].

When multiple polymer types are used in the matrix, their compatibility must be ensured as well in order to achieve optimal performance of the PNC [13, 17, 18]. For example, in the study of Chen and Ahmad [17], the compatibility of non-polar HDPE and polar PET improved with the presence of copolymers. Similarly, in the study of Garofalo et al. [13], use of maleic anhydride (MAH) as a compatibilizer enhanced compatibility of waste PE and PP present in the matrix, and hence improved nanomaterial dispersion and the distribution of nanoclay. However, it is notable that compatibilization of polymer blends is challenging due to differences in polymers’ degradation behavior as well because of their unknown lifetime history as a plastic product [13].

## Processing of polymer nanocomposites

To maximize the reinforcing effect and to achieve the desired properties, nanomaterials must be uniformly distributed in polymer matrix [10]. However, dispersion is challenging when material dimensions are at nanoscale because of the extremely small distances between the nanoparticles, which leads to large van der Waals or electrostatic interactions between the particles, making nanomaterials dispersal into single particles difficult. Therefore, the main challenge in PNC processing is to prevent agglomeration and to ensure proper nanomaterial dispersion in the polymer matrix [9, 12]. Another challenge in PNC processing is addressing the increased viscosity of polymer melt caused by nanomaterial addition [12].

Common methods to disperse nanomaterials into a virgin polymer matrix are melt blending, solution mixing, shear mixing, three-roll milling, ball milling, and in situ polymerization [9, 10]. Common dispersion methods in the plastic waste nanocomposites observed in the current work have been melt blending with a twin-screw extruder [8, 13, 17, 19, 24–26, 29, 39, 41, 42], two-roll milling [20–22, 43], solvent blending [10, 23, 31, 33, 38], and electrospinning [27, 32, 35–37, 45, 46], as well as other methods such as in situ polymerization [30]. Different processing methods are used as a secondary processing step to shape plastic waste nanocomposites into solid test specimens, foams, or nanocomposite fibers. For example, electrospinning and solution blowing spinning (SBS) are used to process nanocomposite fibers, whereas solid nanocomposites suitable for mechanical testing are shaped via injection and compression molding, and by extrusion.

Drying is an important step in plastic waste preparation because moisture has a negative impact on the manufacturing processes and the properties. Therefore, washed plastic waste is often dried before nanocomposite processing [25]. Belioka et al. [26] washed, dried, and cut PET and PLA bottle waste before NC processing with nanoclay. Satya and Sreekanth [10] collected HDPE plastic waste based on resin code, and washed, dried, and extruded the waste into pellets. Yasin et al. [28] prepared PET plastic waste cups by washing them with deionized water, followed by drying, before electrospinning. Malikov [48] dried, ground, and passed plastic waste of cable ties through the sieve to obtain polymer powder, followed by sonification to minimize the grain size. The powder was then washed several times, filtered, and finally dried.

PET waste is currently recycled mainly via mechanical recycling [29]. However, mechanical recycling can cause a drop in the molecular weight of PET and degrades its properties, making it unsuitable for value-added applications [29, 30]. In addition to mechanical recycling, PET waste can be chemically recycled, where, for example, aminolysis reaction of PET waste can be utilized to produce terephthalamide monomers [29]. Lee et al. [29] chemically processed plastic waste into a thermoplastic polyamide elastomer (TPAE) by deriving a renewable bis(6-aminohexyl)terephthalamide (BAHT) monomer through aminolysis of PET waste and combining it with CNTs in a melt blending process to synthesize polymer nanocomposite foam. Shamsi et al. [30] prepared PET bottle waste into polyurethane (PU), after which CNTs were introduced via in situ polymerization. Mallakpour and Behranvand [31] converted PET bottles into white fine powder by heating in a solution, followed by cooling with distilled water, after which functionalized MWCNTs were introduced via an ultrasonic irradiation-assisted solution method.

### Melt blending with a twin-screw extruder

Nanomaterials can be dispersed into a polymer matrix using a twin-screw extrusion process, which is known as the melt blending method. Polymer pellets or powder can either be first combined with nanofillers at high temperatures to fabricate a master batch or both materials can be fed straight into the extruder. Nanomaterial loading and processing temperature also influence the dispersion as high loading causes high viscosity and torque, which are challenging in respect to processing. High processing temperature can reduce viscosity but, on the other hand, may degrade polymer or nanomaterial properties. The challenge with nanomaterial dispersion using twin-screw extruder is the large physical gap between the screws compared to the nanoparticles’ sizes, which can result in a failure to disperse the nanomaterials into individual particles, leading to agglomeration [9].

Plastic waste mechanical recycling using melt processing created also challenges because of the presence of different polymer grades, additives, and other materials in the plastic waste stream, which are difficult to separate from each other, creating polymer blends rather than formation of single polymers [5, 55]. Polymers in recycled blends are often immiscible and incompatible which each other having also different melting temperatures, leading often to formation of recycled polymer blend with poor properties [55]. The use of compatibilizers can improve the compatibility between polymers, however, choosing the optimum processing temperature for polymer blends is a challenging task because high temperature might cause thermomechanical degradation to one polymer while the other polymer in the blend remains still in solid state [5, 55].

Rigail-Cedeño et al. [18] prepared PNC from waste-derived HDPE and PP with ratios of 83 wt% and 15 wt%, respectively, and reinforced the polymer blend with 3 wt% organoclay with and without a compatibilizer using melt blending. Compatibilizer enhanced the nanoclay intercalation and decreased nanocomposite’s viscosity thus improving the processing characteristics in the extrusion process. Chen et al. [56] prepared a polymer nanocomposite from rHDPE/rPET blend combined with compatibilizer and nanoclay using a co-rotating twin-screw extruder. They observed that two-step blending was more effective compared to one-step blending in respect to enhanced clay dispersion into the polymer blend matrix. In two-step blending, recycled polymers and compatibilizer were first prepared into a masterbatch using extruder, followed by crushing and further extrusion with nanoclay, whereas in one-step blending all the materials were combined together in a same extrusion process [56].

Garofalo et al. [13] grafted plastic waste flakes containing mainly PE with a small share of PP in extrusion process with MAH as a compatibilizer. The polymer material obtained was cooled in water, followed by pelletizing after being fed once again into the twin-screw extruder with 5 wt% of organoclays. Similarly, Belioka et al. [26] mixed rPET with rPLA followed by extrusion, to obtain polymer blend pellets that were then melt compounded with two types of nanoclays using twin-screw extrusion.

Istrate and Chen [24] used a twin-screw extruder to combine waste-derived PE, polyethylene-grafted-maleic anhydride (PEgMA) as compatibilizer, and 4 wt% nanoclay. The materials were passed through the extrusion process three times to ensure proper blending. After extrusion, the cooled pellets were shaped with an injection molder to obtain test specimens for tensile and impact tests. In the study, waste-derived PP was similarly melt-compounded with nanoclays without compatibilizer [24].

In the study of Nor Arman et al. [8], HDPE and PET waste were manually mixed with nanoclays or MWCNTs after which they were melt-blended in a twin-screw extruder. The nanocomposite pellets produced were processed in a compression molding equipment using a hot and cold pressing process to obtain test specimens. In similar manner, Chen and Ahmad [17] melt-mixed recycled polymer blend consisting of HDPE and PET with nanoclay, rice husk flour, and a compatibilizer in a co-rotating twin-screw extruder, followed by cooling, granulation, and compression molding.

Velásquez et al. [39] produced a homogenized polymer blend consisting of virgin and recycled PP before adding it into a hopper with 1, 3 and 5 wt% of nanoclay. Melt blending was carried out as cast-extrusion in a twin-screw extruder to obtain PNC films. A similar processing method was used in the studies of Velásquez et al. [25] and Velásquez et al. [41], where nanocomposite films were synthesized from an rPET/vPET blend reinforced with two different nanoclays and from PP waste reinforced with nanosilica, respectively.

In the study of Ahmadian Hoseini et al. [44], melt blending was carried out with a mixer, followed by compression molding. Plastic waste foam consisting mainly of vulcanized nitrile butadiene rubber and recycled PVC was used as a polymer matrix and combined with CNTs to fabricate polymer nanocomposite samples. Similarly, Ismail et al. [40] combined recycled PP and coconut shell waste milled to nanoscale with a rheomixer followed by extrusion. Samples were prepared with compression molding.

Polymer foaming is a technique to create materials with low density and good insulating properties [19]. Oide et al. [19] used recycled HDPE and EVA with 70/30 wt%, respectively, and 2 wt% of nanoclay to produce PNC pellets with a twin-screw extruder. Nanocomposite foam was then prepared from pellets in a flat die single-screw extruder in the presence of 1.5 wt% endothermic foaming agent. In the study of Lee et al. [29], nanocomposite foam was prepared from PET waste derived thermoplastic polyamide elastomer (TPAE), single-walled carbon nanotubes (SWCNT) and MWCNT. Nanomaterials were compounded separately with a TPAE matrix via twin-screw extrusion. Nanocomposite foam was fabricated using microcellular foaming.

**Fig. 1:**
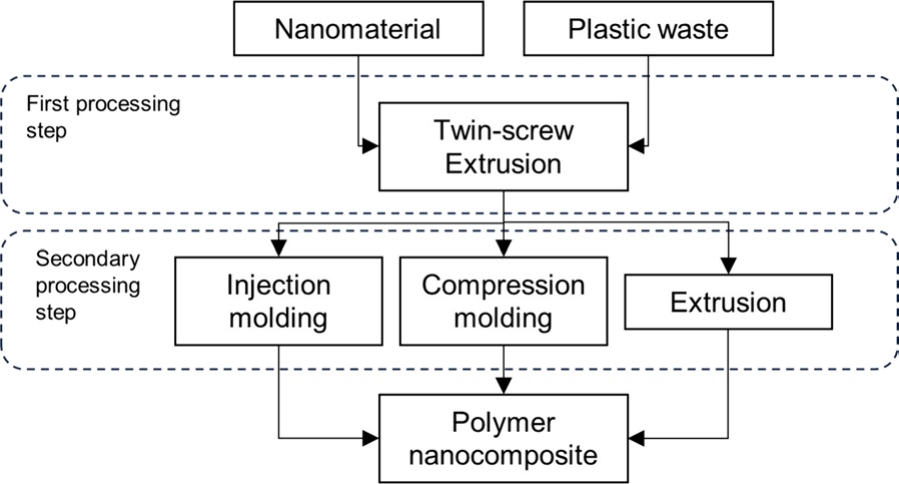
Twin-screw extrusion as a dispersion method to process plastic waste nanocomposites

To summarize, twin-screw extrusion is a common melt blending method to disperse nanomaterials, mainly nanoclays but also nanosilica and CNTs, in a plastic waste matrix. Typically, plastic waste and nanomaterials are fed into an extruder with or without a compatibilizer. The extrusion process can be carried out multiple times to enhance the nanomaterial dispersion, after which the pellets obtained can be shaped into solid test specimens in a secondary processing step via injection or compression molding or processed into a foam structure in an extrusion process using a foaming agent. Typical melt blending procedures to synthesize polymer nanocomposites from plastic waste via twin-screw extrusion as a dispersion method are presented in Fig. 1.

### Two-roll mill mixing

In roll milling, materials are passed multiple times through rotating rollers. Shear forces are generated allowing nanomaterials to mix and disperse into the polymer matrix. The main advantage of roll milling is that materials with high viscosity can be processed due to the high shear forces, meaning that high nanomaterial loadings can be used. On the other hand, the gap between the rollers is at least one micrometer in size, making it impossible to disperse nanomaterials into individual particles [9].

Anto and Rejeesh [20] fed waste HDPE flakes between the rollers of a two-roll mill mixer using 150 °C as the operating temperature. Aluminum oxide nano powder was introduced into the process, after which the material produced was compression molded. In similar manner, Mahmoud et al. [21] prepared rHDPE polymer melt in a two-roll mill mixer at 180 °C, followed by dispersion of copper oxide NPs and phosphotungstic acid (PTA) into the mixer. When the material was homogenous, it was milled, followed by compression molding. Using a similar processing technique, El‐Taher et al. [22] and El-Sharkawy et al. [43] prepared PNCs from HDPE waste, nano copper oxide and PTA, and PVC waste and bismuth oxide (Bi_2_O_3_) NPs, respectively.

**Fig. 2:**
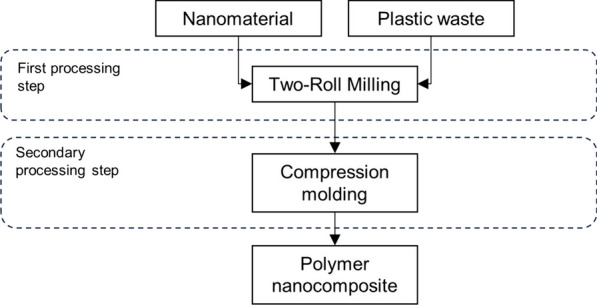
Two-roll mill mixing as a dispersion method to process plastic waste nanocomposites

Based on study of published literature, two-roll milling, followed by compression molding can be used to obtain plastic waste nanocomposites for radiation shielding applications from aluminum and copper oxide NPs with HDPE waste, and bismuth oxide NPs with PVC waste. A typical process to synthesize plastic waste nanocomposites via two-roll milling is presented in Fig. 2.

### Solvent blending

Solvent blending, also known as solution mixing and solution dispersion, is a straightforward method to produce polymer nanocomposites. Nanomaterials are first dispersed into a solvent, followed by mixing with the polymer solution. To ensure proper nanomaterial dispersion and to prevent agglomeration, mixing is often carried out through ultrasonification [9]. Solvent blending can be also conducted by first preparing the polymer solution followed by the addition of the nanomaterials [57]. After proper mixing of the polymer-nano solution, the solvent is evaporated and dried, followed by extrusion or injection molding to obtain solid polymer nanocomposite samples.

Satya and Sreekanth [10] prepared polymer nanocomposites via a solvent blending method. First, 0.1, 0.3 and 0.5 wt% of graphene and MWCNT were mixed with ethanol to prepare a nanofluid, followed by ultrasonication assisted blending. HDPE waste pellets were then immersed into the nanofluid and heated up until the solvent evaporated. The nanomaterial coated pellets obtained were then dried in an oven to remove moisture, after which the pellets were melt-blended with a twin-screw extruder, followed by injection molding to ensure uniform dispersion. Similarly, Reddy et al. [23] dispersed from 0.25 to 4 wt% graphene flakes into toluene by sonification, after which chopped HDPE waste was added to the mixture, followed by sonification-assisted mixing. The mixture was stirred, and the solvent evaporated. Finally, the PNC samples were processed with an injection molder.

Mallakpour and Behranvand [31] dispersed 1, 2 and 4 wt% of D-glucose functionalized MWCNTs via sonification in solution. First, PET waste was mixed with solvent, followed by addition of MWCNTs into the mixture. After proper mixing, the solvent was removed by drying at 120 °C for 24 h to obtain a solid PNC material.

**Fig. 3:**
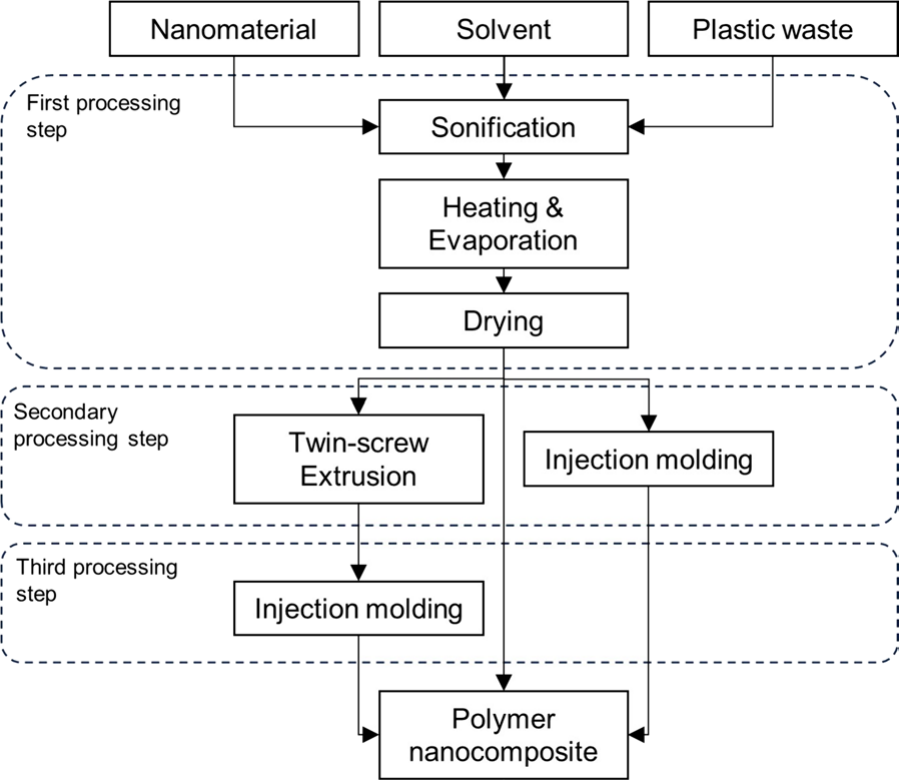
Solvent blending as a dispersion method to process plastic waste nanocomposites

Study of the literature indicates that mainly carbon-based nanomaterials are combined with plastic waste via the solvent blending method. Typically, plastic waste pellets or flakes are added into nanofluid, followed by evaporation of the solvent, after which the nanocomposite produced is dried in an oven to remove remaining moisture. Extrusion and injection molding are used as secondary processing steps to shape the samples. A typical process to synthesize PNCs with solution blending from plastic waste is presented in Fig. 3.

### Electrospinning and solution blowing spinning

Electrospinning and solution blown spinning (SBS) are methods to produce polymer nanofibers with a diameter in the nanoscale. Electrospinning is a relatively common processing method whereas SBS is a recently developed technique [28, 47].

In the studies of Uddin et al. [45] and Uddin et al. [46], chopped EPS waste was dissolved with dimethylformamide (DMF) and acetone, followed by stirring at 40 °C for a few hours after which titanium dioxide NPs and aluminum microparticles were added into the polymer solution. After sonification to ensure proper nanomaterial dispersion, the solution was transferred to a container attached to the needle of electrospinning equipment. After nanofibers were produced, they were dried on a screen for 24 h, followed by drying in the oven to remove residual solvent and trapped moisture. Similarly, Asmatulu et al. [35] used an electrospinning process to prepare nanocomposite fibers from PVC and PS waste blended with nickel zinc ferrite (NiZnFe_2_O_4_) nanoparticles. First, chopped PS and PVC were dissolved in DMF, followed by addition of nanoparticles into the solvent via 30 min of sonification. Before the electrospinning process, the solution was stirred with a magnetic bar for 12 h. de Oliveira Santos et al. [27] prepared a solution containing waste PET, castor oil and cellulose nanocrystals (CNC) and used electrospinning to fabricate composite mats with randomly, aligned, and non-aligned orientations.

Khan et al. [37] prepared nanocomposite fibers from PS waste, MWCNTs and NiZn ferrite NPs using an electrospinning process. First, the nanomaterials were dispersed into DMF as solvent and sonicated for 30 min, followed by addition of chopped PS pieces. The mixture was agitated at high speed at 50 °C for 12 h before electrospinning. Nanocomposite fibers were collected after drying for 24 h at room temperature, followed by drying in an oven to remove residual solvent and trapped moisture.

Unlike in other electrospinning studies considered in this review, Yasin et al. [28] first converted PET waste into nanofibers in an electrospinning process, followed by addition of copper oxide (CuO) nanoparticles on the surface of PET nanofiber using a cross-linked solvent. Soltanolzakerin-Sorkhabi et al. [32] prepared nanofibrous membranes from waste PET solution by electrospinning, which was followed by NaOH modification to assist the decoration with silver NPs using in situ chemical green synthesis. Mogharbel et al. [47] created colorless polymer out of waste PC using a refining process followed by mechanical stirring with 20 wt% of pure PC, 1.5 wt% of plasticizer and up to 1.5 wt% of strontium aluminate NPs in DMF. The produced solution was charged into the SBS equipment to fabricate nanofibrous membranes.

**Fig. 4:**
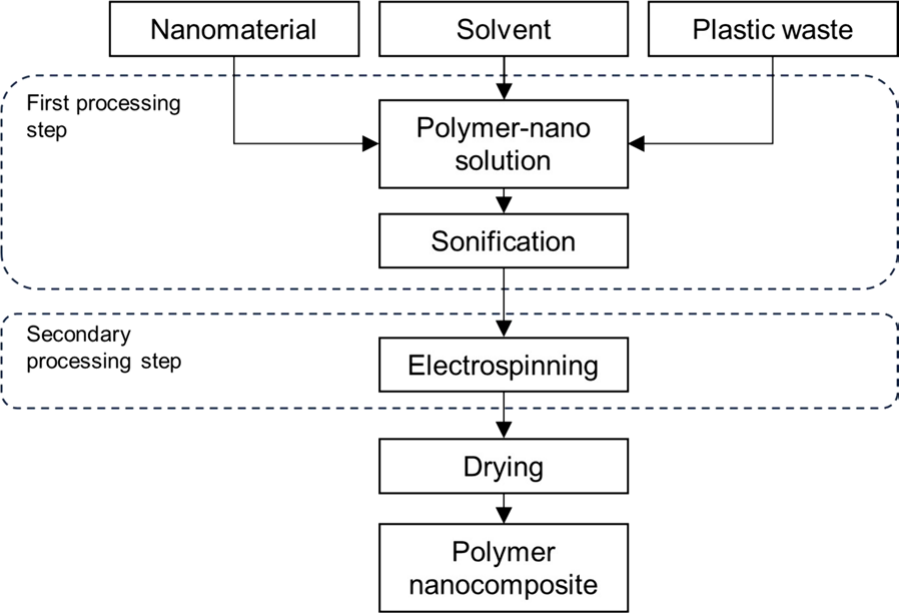
Electrospinning as a processing method to produce nanocomposite fibers from plastic waste

Based on the papers reviewed, it can be seen that different plastic waste grades–PET, PS, PVC and EPS, and various nanomaterials, including MWCNTs, cellulose nanocrystals, and copper, NiZn ferrite, titanium dioxide, and silver nanoparticles have been processed by electrospinning to produce nanocomposite fibers and mats. The polymer-nano solution is prepared primarily by dissolving polymers into DMF solvent, followed by sonification-assisted nanomaterial inclusion and hours long agitation before electrospinning. Drying is the last processing step to remove remaining solvent. A typical process to prepare polymer nanocomposite fibers from plastic waste using electrospinning is presented in Fig. 4.

### Other processing methods

In addition to extrusion, roll-milling, solvent blending and electrospinning, which form the mainstream, some other processing techniques have also been investigated to combine plastic waste with nanomaterials. Malikov [48] synthesized copper sulfide (CuS) NPs in polymer powder obtained from cable tie waste using a successive ionic layer adsorption and reaction (SILAR) method to produce nanostructure material. Shamsi et al. [30] converted PET waste into PU, followed by nanocomposite synthesis with CNTs via in situ polymerization. Polymer nanocomposite synthesis with in-situ polymerization starts with swelling of nanomaterials in monomer solution, where low-molecular-weight monomer penetrates between interlayers of single nanoparticle. This step is followed by polymerization using heat, radiation, or an initiator to create an intercalated or exfoliated structure [58]. Naguib [34] prepared PNC samples from PE waste and magnesium hydroxide NPs, where nanomaterials were dispersed into the matrix using mechanical stirring followed by sonification in an ice bath. Finally, the material obtained was cured with 1 wt% of curing agent. This processing method was described as in situ polymerization curing [34].

All in all, while reviewed plastic waste nanocomposite publications report manufacturing processes being successful in respect to improvement in the plastic waste morphology and properties, there is a lack in research in the evaluation of the synergistic effects of nanomaterials and plastic waste for processability. Further research in plastic waste NC processing could also be focused on optimization of the processing parameters, especially with contaminated plastic waste steams having polymers with different melting temperatures and characteristics.

## Properties and characteristics of plastic waste nanocomposites

As observed in previous studies and reported by Nor Arman et al. [8], nanomaterial addition can influence multiple properties of virgin and recycled polymers including barrier effect, flame retardancy, thermal stability, and electrical and thermal conductivity. The following sections present the effect of various nanomaterials on the morphology, and mechanical, thermal, physical, and electrical properties of plastic waste nanocomposites.

### Morphology

The reinforcing effect of nanomaterials and improvement in the strength properties of PNCs are directly correlated with the improved matrix-nanomaterial interaction [17]. In general, agglomeration of nanomaterials is not desired as it often leads to inferior mechanical and thermophysical properties [26]. Therefore, large amounts of nanoclay and most other nanomaterials should be avoided as it often leads to agglomeration [26]. Additionally, the type of nanoclay has an effect on the nanomaterial dispersion, as shown in the study by Istrate and Chen [24], where the use of organomodified nanoclay resulted in an intercalated nanostructure and good dispersion in a recycled polymer matrix, whereas the use of natural nanoclay presented mainly as aggregates.

In the study of Istrate and Chen [24], PS waste-based nanocomposite showed an intercalated and exfoliated structure, whereas waste-derived compatibilized PE formed only conventional composites when neat and blowing agent treated nanoclays were added via melt blending. Impurities and too little amount of compatibilizer might have influenced the poor interface between the clay minerals and the recycled matrix, leading to conventional composite morphology. An exfoliated structure was observed also in the study of Oide et al. [19], where nanocomposite foam was synthesized from an rHDPE/rEVA blend and nanoclay. Rigail-Cedeño et al. [18] reinforced the rHDPE/rPP polymer blend with 3 wt% organoclay with and without a compatibilizer using extrusion process. An intercalated structure was observed, which was enhanced by the presence of compatibilizer. Velásquez et al. [25] prepared nanocomposite films from an rPET/vPET blend reinforced with nanoclay. An intercalated structure was observed when the rPET content was higher than the vPET content, which was explained by the shorter chains of rPET, which favor the polymer intercalation. In contrast, a tactoid structure with more agglomeration was observed when the vPET concentration in the polymer blend was greater than the rPET concentration.

In the study of Belioka et al. [26], lower amounts of clay in a rPET/rPLA polymer blend exhibit partially exfoliated and intercalated structure, while higher clay content (10 wt%) showed only an intercalated structure with aggregates. Similar observations were made in the studies of Velásquez et al. [41] and Nor Arman et al. [8], where nanomaterial dispersion into recycled matrices was better with lower nanomaterial content and increased amounts of nanomaterial led to agglomeration and deterioration in properties. Interestingly, when bismuth oxide (Bi_2_O_3_) nanoparticles with ratios of 5, 15, 25, 35 wt% were dispersed into recycled PVC via melt blending, it was observed that the nanoparticles were dispersed homogenously into the recycled matrix with loading up to 35 wt% [43].

In summary, good nanomaterial dispersion into the polymer matrix while avoiding agglomeration has a significant effect on improvement in the properties of the plastic waste. Use of a suitable compatibilizer can enhance the nanomaterial dispersion and morphology, further improving the properties of plastic waste. Conversely, impurities in the plastic waste, too little compatibilizer, and high nanomaterial content can cause agglomeration, limiting property improvements.

### Mechanical properties

Improvement in the mechanical properties of plastic waste is desired as it widens the range of possible applications of the secondary material. In the study of Oide et al. [19], mechanical and thermal properties and the degree of crystallinity of a recycled polymer blend were enhanced by nanoclay inclusion. Mechanical properties of plastic waste were also found to improve in the study of Shamsi et al. [30] when CNTs were added into PET waste derived PU.

In the study of Rigail-Cedeño et al. [18], adding nanoclay into a recycled HDPE blend with small share of rPP increased stiffness, strength, and toughness of the rHDPE/rPP blend. However, the presence of compatibilizer decreased stiffness of the nanocomposite. Similarly, in the study of Istrate and Chen [24], the presence of nanoclay in waste PE and PS matrices improved stiffness of the NC compared to neat rPE and rPS. The blowing agent treated clay doubled the impact strength of the PS waste compared to the nanocomposite prepared from PE waste. Addition of clay into compatibilized rPE matrix significantly improved the energy at break value, explained by the obtained intercalated structure [24]. In the study of Garofalo et al. [13], the stiffness and elasticity of plastic waste containing mainly PE and a small share of PP was improved as a result of nanoclay inclusion, the latter one explained by the improved compatibility between different polymers present in the matrix gained by nanomaterial addition. Use of maleic anhydride (MAH) enhanced also the compatibility between the polymers while improving the nanomaterial dispersion and doubled the elongation at break value compared to neat plastic waste [13]. Work carried out by Nor Arman et al. [8] showed that the presence of MWCNT and nanoclay significantly increased tensile properties of a recycled polymer blend consisting of HDPE and PET with a weight ratio of 75/25, respectively. The significant increase in young’s modulus with nanomaterial inclusion was explained by the interfacial bonding between the clay particles and polymer chains, and the improved interfacial adhesion of the polymer blend, compared to neat matrix. The optimum nanomaterial loading with respect to tensile properties was lower with nanoclay than with MWCNTs. Impact strength of the rHDPE/rPET blend decreased with increase in both nanomaterial loadings, although the decrease was more dramatic with MWCNT [8].

In the studies of Velásquez et al. [41] and Velásquez et al. [42], recycled PP were reinforced with nanosilica and nanoclay, respectively, to produce nanocomposite films. Both nanomaterials improved the elasticity and tensile strength of the recycled polymers. Nanosilica also improved the seal strength of the nanocomposite films although decreasing elongation at break value, compared to rPP. The best mechanical performance was achieved with 1 wt% nanosilica inclusion. Interestingly, the presence of nanoclay in the rPP did not change the elongation at break value when the nanomaterial loading was below 2 wt%. It was noted that elongation at break started to decrease with nanosilica inclusion above 2 wt%. In the study of Velásquez et al. [39], adding nanoclay into a polymer blend consisting of 50 wt% virgin and 50 wt% recycled PP improved tensile strength, elasticity, and barrier properties but reduced ductility and elongation at break, compared to rPP. It was observed that the best mechanical and thermal properties were gained with 1 wt% inclusion of more hydrophobic non-polar-modified nanoclay. Higher nanomaterial loading reduced tensile strength and elasticity due to agglomeration of the nanomaterial. In the study of Velásquez et al. [25], the presence of nanoclay in a polymer blend with a higher concentration of rPET than vPET increased elasticity of the polymer blend due to the presence of shorter polymer chains in the recycled polymer.

In the literature review carried out by Zare [11], it was concluded that the highest stiffness and strength of the rPET/nanoclay composite is achieved with higher nanomaterial content, while optimum levels of strength and toughness are gained with lower content (2 wt%) because nanoclay reduces the mobility of polymer chains. Ismail et al. [40] prepared fully waste-derived nanocomposites from PP waste, and coconut shell waste milled at nanoscale. The study revealed that the optimum performance of the nanocomposite was achieved with a 30/70 wt% plastic waste to coconut shell ratio, which is an unusual ratio for the nanomaterials.

In summary, mechanical properties such as tensile strength, stiffness, toughness, and elasticity of plastic waste can be improved with low loadings of different nanomaterials. Optimum mechanical properties are obtained with low nanomaterial loadings as higher loadings lead to poor dispersion and agglomeration, limiting improvement in properties. Based on the papers reviewed, addition of nanomaterials can work as a compatibilizer between different polymers present in the plastic waste matrix thus improving the mechanical properties.

### Thermal properties and flame retardancy

The thermal stability of a polymer matrix can be improved by nanomaterial inclusion due to a barrier effect [54]. According to Chen and Ahmad [17], the degree of exfoliation of nanoclay is the key factor affecting the flame retardancy of polymer nanocomposites. Thermal stability, flame retardancy and mechanical properties of a recycled polymer blend were enhanced in the study of Chen and Ahmad [17] by use of nanoclay and rice husk inclusion due to enhanced compatibility between the non-polar HDPE and polar PET phases, intercalation of the clay, and increased interfacial adhesion between the nanomaterials and recycled polymer blend. In the study of Naguib [34], the thermal stability and flame retardancy of waste PE was improved by inclusion of magnesium hydroxide nanoparticles. The most effective flame retardancy and improved hardness were achieved with 15 wt% nanomaterial addition [34]. Similarly, aluminum oxide nanoparticles improved thermal stability of rHDPE while providing shielding efficiency for HDPE waste in the study of Anto and Rejeesh [20].

In the study by Velásquez et al. [39], adding nanoclay into a polymer blend consisting of 50/50 wt% virgin and recycled PP, respectively, improved the thermal performance of the waste material. The greatest improvement in rPP properties was obtained using a hydrophobic modified nanoclay. In similar manner, the study of Belioka et al. [26] demonstrated that the presence of nanoclay enhanced the thermal stability of a recycled polymer blend (rPET/rPLA), and the increased amount of clay increased the thermal degradation, compared to neat polymer blend. Additionally, higher amounts of PET compared to PLA provided better thermal stability. Similar results were found in the study by Satya and Sreekanth [10], where thermal stability, thermal distortion temperature and thermal degradation temperature of rHDPE were improved by increasing the loadings of graphene and MWCNT. Optimum nanomaterial loading for thermal applications was found to be 0.5 wt%, while higher loading also improved interfacial bonding, thus enhancing the strength properties of the rHDPE. Samples with 0.3 wt% and 0.5 wt% of MWCNT and graphene, respectively, showed improved stiffness and strength compared to neat rHDPE, indicating viscoelastic behavior. Samples with 0.3 wt% nanomaterial loading showed also good load bearing capacity [10].

In the study of Istrate and Chen [24], the thermal degradation temperature of an rPS-based nanocomposite increased marginally with nanoclay reinforcement but decreased when rPE was reinforced with nanoclay, compared to neat polymers, explained by a dominating catalytic effect of the surfactant. Mallakpour and Behranvand [31] prepared a polymer nanocomposite from recycled PET bottle waste and MWCNTs using a solvent blending method. Compared to properties of PET waste, crystallization of the recycled PET increased with the presence of MWCNTs as a nucleating agent while the melting point decreased due to weakening of the intermolecular interactions between the polymer chains caused by interaction of the MWCNTs with the polar polymer matrix [31].

Asmatulu et al. [35] used an electrospinning process to produce nanocomposite fibers from PVC and PS waste with incorporation of NiZn ferrite nanoparticles. The study revealed that the addition of the magnetic nanomaterials improved the thermal conductivity of the plastic waste. Likewise, Khan et al. [37] prepared nanocomposite fibers from waste-derived PS reinforced with MWCNTs and NiZn ferrite NPs using an electrospinning process. The study showed that the nanomaterials enhanced the thermal conductivity, dielectric constant and superhydrophobic properties of the rPS fibers. Also, adding ferrite-based NPs resulted in superparamagnetic behavior.

In summary, the thermal properties of plastic waste can be improved by inclusion of different nanomaterials, and higher nanomaterial loading enhances thermal stability of recycled polymers further. Improvement in flame retardancy is observed when using nanoclay and magnesium hydroxide nanoparticles, and improvement in thermal conductivity is found with MWCNT and NiZn ferrite nanoparticles, based on the papers reviewed.

### Physical properties

In the study of Nor Arman et al. [8], the density and percentages of water uptake in an rHDPE/rPET blend was found to increase with the inclusion of increased amounts of MWCNTs and nanoclay, although values were still relatively low and at acceptable levels. Velásquez et al. [41], Velásquez et al. [42], and Velásquez et al. [39] investigated barrier properties of nanomaterials combined with recycled polymers for food packaging applications. The water vapor and oxygen permeability of the waste PP did not change with the presence of 1 wt% nanosilica by Velásquez et al. [41]. On the other hand, nanoclay was found to decrease the water vapor permeability and increase oxygen permeability of waste PP in Velásquez et al. [42] study.

The overall migration limit defines the maximum permitted number of substances used in plastic packaging that can be released from packaging material into a food simulant. The current value in EU regulations on food contact plastics is 10 mg of substances per 1 dm^2^ [59]. In Velásquez et al. [41] and Velásquez et al. [42], the limit for aqueous and fatty simulants of PP waste nanocomposites were not exceeded when using nanoclay, but exceeded when nanosilica was used for nanocomposite fabrication. However, the presence of nanosilica reduced the overall migration of rPP due to a barrier effect provided by the nanomaterial [41]. In Velásquez et al. [39], adding nanoclay in an rPP/vPP polymer blend improved not only the barrier effect against the fatty simulant but also enhanced the thermal stability and improved some mechanical properties. The type of clay had an effect on the physical properties of the polymer blend as the hydrophobic-modified nanoclay provided higher thermal stability and greater intercalation but increased overall migration to the fatty simulant compared to nanoclay with a more hydrophilic nature. Notable is that the nanoclay did not appear to affect migration into 3% acetic acid and 10% ethanol food simulants.

de Oliveira Santos et al. [27] used electrospinning to fabricate composite mats with different orientations from waste PET solution mixed with castor oil and cellulose nanocrystals. The castor oil worked as a biodegradable and non-toxic compatibilizer between the recycled matrix and the nanomaterial, increasing elasticity and tensile strength compared to nanocomposite mats without a compatibilizer. In addition, mats with nonaligned orientation exhibited hydrophilic behavior, while aligned arrangement resulted in a hydrophobic surface. Asmatulu et al. [36] prepared superhydrophobic nanocomposite fibers from waste PVC and PS with MWCNTs and NiZn ferrite NPs. The presence of the nanomaterials improved the contact angle value of the plastic waste, indicating a superhydrophobic surface. Uddin et al. [45] and Uddin et al. [46] prepared superhydrophobic nanocomposite fibers from recycled expanded polystyrene (rEPS) foam, titanium dioxide nanoparticles and aluminum microparticles. The studies revealed that inclusion of the nanomaterial improved the superhydrophobic property and thermal stability of the waste material. In the study of Mogharbel et al. [47], solution blowing spinning was used to prepare nanocomposite fibers from PC waste and strontium aluminate nanoparticles. Colorless fibers showed photochromism to green under UV light. Increased nanomaterial content provided better superhydrophobicity, and the best photochromic behavior was observed with 1 wt% nanomaterial loading.

In summary, nanomaterials provide a barrier effect for plastic waste. In respect to food simulants, nanoclay provides a more efficient barrier than nanosilica. The use of nanomaterials, MWCNTs and nanoclay, increase slightly the density and water uptake of plastic waste. Superhydrophobic nanocomposite fibers can be synthesized from plastic waste by electrospinning with inclusion of different nanomaterials.

### Electrical and shielding properties

El‐Taher et al. [22] and Mahmoud et al. [21] prepared effective shielding materials for gamma and X-ray radiation from recycled HDPE, copper oxide nanoparticles and PTA. The shielding property of the rHDPE was enhanced with the presence of PTA and copper oxide NPs due to their small particle size and the high bulk density of PTA. It was noted that the electron density, mass attenuation coefficient, and effective atomic number of waste HDPE increased to a greater extent with PTA than when using copper oxide NPs [21]. El-Sharkawy et al. [43] prepared gamma radiation shielding material from recycled PVC and bismuth oxide (Bi_2_O_3_) nanoparticles via a melt blending method. The study revealed that bismuth oxide NPs provided shielding efficiency for rPVC, and the mass coefficient increased significantly with increased nanoparticle content.

Nanocomposite foam from PET waste derived TPAE and CNTs showed favorable conductivity, high specific shielding effectiveness and excellent durability with the presence of only 2 wt% of SWCNT, compared to properties of plastic waste. In addition, the foam was found to be easily recyclable with a possibility for reprocessing [29]. Reddy et al. [23] prepared electrically conductive nanocomposite from waste HDPE and graphene. The nanomaterial addition improved dielectric properties of rHDPE only slightly because of the non-polar characteristic of the HDPE. On the other hand, the electrical conductivity of the rHDPE was significantly improved by the presence of graphene due to polarization and electron mobility at room temperature.

Ahmadian Hoseini et al. [44] used melt blending to prepare electrically conductive nanocomposite from waste foam consisting of vulcanized nitrile butadiene rubber and waste PVC reinforced with CNTs. The plastic waste was first mixed with virgin PS as a binding material because the plastic waste was not meltable due to the high rubber content. CNTs were then added into prepared polymer matrix. Electrical conductivity and EMI shielding properties were improved by the presence of plastic waste compared to virgin PS/CNT nanocomposite. Xie et al. [49] prepared an electrically and thermally conductive EMI shielding nanocomposite material from aluminum-plastic packaging waste combined with graphite flakes via a microwave-assisted sintering process. The study showed that the sintering process provided better properties for the composite compared to use of hot molding due to exfoliation and in-situ reduction of graphite.

To summarize, the electrical and radiation shielding properties of plastic waste can be enhanced with low loadings of carbon-based and metallic nanomaterials. The use of compatibilizer can also improve the radiation shielding efficiency of plastic waste.

## Applications for plastic waste nanocomposites

Value-added materials can be prepared from plastic waste via nanomaterial reinforcement, because of the improvement in the plastic waste properties. However, nanomaterials used should be selected according to the specific application of interest as different nanomaterials affect the properties of plastic waste nanocomposites in different ways [54].

### Water treatment and filtration systems

Yasin et al. [28] found that adding copper NPs in the surface of rPET nanofibers provided high photocatalytic activity for methylene blue dye removal. Janqamsari et al. [33] prepared efficient sorbent material, a nanocomposite aerogel, from rPET fibers, PVA and CNTs for oil spill removal from water. Ghambari et al. [38] prepared a magnetic nanocomposite from PS waste and cobalt ferrite NPs for dispersive micro-solid phase extraction of selected parabens from water samples.

In the study of de Oliveira Santos et al. [27], PET waste, castor oil and cellulose nanocrystals containing electrospun mats showed potential for filtration and biomedical applications. Likewise, electrospun nanofibrous membranes from PET waste decorated with silver NPs showed good antimicrobial and antibiofilm activity, making it a promising material for eradication of microbes, wound dressings, air filters, and water purification applications [32].

Nanocomposite fiber synthesized from PVC waste, MWCNTs and NiZn ferrite NPs showed high surface area with superhydrophobic behavior suitable for various industrial applications including filtration, biomedical engineering, defense, textile, and aerospace [36]. Likewise, Uddin et al. [45] and Uddin et al. [46] prepared superhydrophobic nanocomposite fibers suitable for various industrial applications such as water collection, water filtration and tissue engineering from rEPS, titanium dioxide NPs and aluminum microparticles.

### Food packaging

Velásquez et al. [41] and Velásquez et al. [42] studied the possibility of using PP waste with nanomaterial reinforcement as a packaging material for food contact applications. In general, adding nanosilica improved the mechanical properties and overall migration of the prepared NC films. However, overall migration to a fatty simulant exceeded the limit value determined in the European Union’s regulation on food contact plastics (10/2011). Adding nanoclay increased oxygen permeability and mechanical properties but decreased water vapor permeability. The overall migration limit for aqueous and fatty simulants was not exceeded when using nanoclay with a recycled polymer matrix. In other work, Velásquez et al. [39] studied the possibility of utilizing nanoclay in an rPP/vPP matrix for food packaging films. It was observed that the sample with hydrophobic modified nanoclay provided higher thermal stability and greater intercalation but higher overall migration to the fatty simulant than nanoclay with a more hydrophilic nature. Overall migration values to 3% acetic acid and 10% ethanol food simulants were below the EU’s limits, although limits were exceeded for migration to a fatty simulant.

According to López de Dicastillo et al. comprehensive review [54], nanomaterials, which of nanoclay seems to be the most promising due to its platelet structure and low cost, can improve the barrier and mechanical properties of packaging material made from plastic waste. However, López de Dicastillo et al. [54] emphasize that the chemical safety and enhancements in properties are dependent on the type of polymer and the processing history of the plastic waste [54]. Plastic waste utilized in above-mentioned studies are obtained as post-consumer recycled PP from flexible PP-based packaging [41, 42], which does not characterize the realistic composition of mixed plastic waste stream. Therefore, the chemical safety of plastic waste nanocomposites in food packaging applications must be considered carefully because of the unknown composition and quality of the realistic plastic waste stream.

### Electronic, shielding, and thermal applications

Lee et al. [29] prepared lightweight nanocomposite foam with excellent EMI shielding performance from TPAE derived from PET waste and CNTs sustainable electronic applications, such as EMI shielding products. Anto and Rejeesh [20] prepared nanocomposite material from rHDPE and aluminum oxide NPs with improved thermal stability and shielding properties suitable for electronic packaging applications.

In the study of Mahmoud et al. [21], CuO NPs and PTA were used with HDPE waste to produce material for gamma and X-ray shielding applications. The material was found to be economically and environmentally advantageous having low thickness, good flexibility, and light weigh, making it suitable as clothing shielding material. El-Sharkawy et al. [43] prepared a sustainable, low cost, lightweight, and flexible gamma radiation shielding material from PVC waste and bismuth oxide (Bi_2_O_3_) NPs. The material was suitable for nuclear energy applications as well as for medical diagnostics and aerospace applications.

Malikov [48] synthesized copper sulfide NPs within a polymer matrix from cable tie (nylon 6,6) waste via successive ionic layer adsorption and reaction method to produce nanostructured material suitable for semiconductor elements, solar panels, and UV protection devices. In the study of Mogharbel et al. [47], UV motivated colorless nanofibrous membranes were prepared for optical anticounterfeiting use from PC waste and strontium aluminate NPs using solution blown spinning (SBS).

Polymer nanocomposites from HDPE waste reinforced with graphene and MWCNTs have shown improved thermal stability, stiffness and strength, making such nanocomposite suitable for thermal management and construction applications [10]. Naguib [34] prepared polymer nanocomposite samples from rPE and magnesium hydroxide NPs with suitability to act as an effective green fire retardant.

To summarize, value-added materials prepared from plastic waste nanocomposites are suggested in literature to be suitable for applications such as food packaging, electronic, shielding, water treatment, medical diagnostics, aerospace, UV protection devices, textiles, thermal management and fire-retardant. Based on the literature reviewed, nanoclay provides the best barrier effect for food packaging purposes, metallic nanoparticles and CNTs are most suggested to be used with plastic waste in electronic, shielding and water treatment applications, and typical applications for electrospun plastic waste nanocomposite fibers can be found in the areas of water treatment and filtration systems. It must be highlighted that most of the suggested plastic waste NC applications are very specific and are not capable to utilize huge volumes of plastic waste. Furthermore, the proposed applications for plastic waste NCs are the same as for virgin PNCs [60], which raises a question of the quality of plastic waste utilized. Therefore, further plastic waste NC research should be focused on to discover applications which are capable to match the high plastic waste volumes and quality of the real plastic waste stream. In addition, safety evaluations must be carefully considered when nanomaterials and waste-derived materials are involved, especially in food contact applications. Also, economic consideration must be taken into account while exploring the potential applications because recycled materials are not always economically comparable with virgin materials due to the additional preparation steps such as plastic waste separation, washing and drying, which increase the costs [7].

## Sustainability and economy of plastic waste nanocomposites

Plastic waste dumped into soil or waterways can leach toxic chemicals such as phthalates into the environment, creating health risks [1]. Also, incineration of plastic waste can liberate hazardous substances such as halogens, dioxins and furans, which cause harm for the environment [1]. The combustion of plastic waste also creates dust, fumes, and toxic gasses[11], if performed in facilities with insufficient exhaust treatment. From this perspective, plastic waste recycling into secondary raw materials seems to be a more promising option than landfilling and incineration, and recycling is also the preferred method in the EU’s waste hierarchy after reduce and reuse [3].

According to Carroccio et al. [53], incorporation of nanomaterials with recycled polymers can be beneficial for the environment due to the replacement of petroleum-based virgin polymers and due to possible material savings achieved through improvement in properties. Light weight gained with material savings influences also on the transportation and usage phases while increasing the sustainability of the manufactured products. On the other hand, even though small amounts of nanomaterials are typically used with polymers, most of the nanomaterials have relatively high environmental impact, CNTs being the worst, because nanomaterial production requires high amounts of energy, leading to high emissions of greenhouse gasses (GHGs) [53]. Organoclays, graphene, and carbon black, on the other hand, have relatively low environmental impact compared to other nanomaterials. Therefore, the advantage of the plastic waste NCs in the environment perspective is mostly dependent on the type of nanomaterial [53].

Even though environmental concerns are important, and sustainability is nowadays supported by national regulations, economic aspects must be also considered. While the use of post-consumer plastic waste in a product can provide advantages from both the environmental and ecological point of view, the cost, quality, and possible applications of plastic waste nanocomposites are highly dependent on the waste type, the nanomaterial used, and the processing method adopted [1, 21]. The additional pretreatments steps for plastic waste stream, including separation and purification, increases the costs and lowers the environmental benefits of the recycled materials [7] because of the water consuming purification and energy-consuming drying. To the best of authors knowledge, plastic waste nanocomposite research is currently limited only to laboratory-scale studies where mainly high-quality plastic waste, such as plastic waste pellets, are utilized, which do not represent the quality of the real plastic waste stream. Therefore, the further research should be focused on the utilization of plastic waste that reflects the realistic condition of the plastic waste stream with minimum purification steps.

According to Ahmadian Hoseini et al. [44], plastic waste NC with improved electrical conductivity and EMI shielding properties from PVC and rubber waste reinforced with CNTs not only turned waste into a value-added product but also reduced the amount of CNTs required to obtain a certain level of conductivity, leading to significant reduction in costs [44]. Additionally, reducing the amount of CNTs and utilizing plastic waste as raw material instead of virgin polymers might reduce not only costs but also be beneficial for the environment.

Nguyen et al. [61] studied energy consumption, GHG emissions and production costs of drainage pipes made from virgin HDPE, a virgin-recycled HDPE blend, and nanocomposite from virgin-recycled HDPE with nanoclay addition. Pipes made of virgin-recycled HDPE blend and the nanocomposite showed environmental and economic advantages over virgin HDPE pipes due to avoidance of energy consuming oil production. GHG emissions of the nanocomposite pipes were over 50% lower compared to virgin HDPE pipes, and 16% lower compared to pipes made of virgin-recycled HDPE blend. Nanoclay production did not significantly affect the environmental burden of the material, and the energy cost of the nanocomposite pipe was mostly affected by processing of the polymer matrix. The nanocomposite was also lighter than the conventional material, reducing labor costs and energy demand during installation and transportation. Overall, the highest cost was found with pipes made from virgin HDPE, whereas lowest production cost was achieved with plastic waste nanocomposite. However, transportation distance of the plastic waste should be considered as long distances leads to increased GHG emissions. Also, Anto and Rejeesh [20] reported that the use of recycled HDPE in nanocomposites is cost-effective way to utilize plastic waste while reducing the eco-footprint.

Circular economy is a model where materials and products are reused and recycled as many times as possible [62]. In practice, no waste is created, and all materials are prepared for reuse after served their intended purpose [62]. While utilizing plastic waste in nanocomposites provides solution to prepare value-added secondary material from waste, plastic waste NC products will receive their end of life as well. Therefore, recyclability of plastic waste NCs is important to consider supporting the circular economy and sustainability. However, to the best of authors knowledge, recyclability of plastic waste NC products have not received attention and should be considered in further research. Virgin PNCs are used in various applications such as in packaging and in the automotive and aerospace industries due to the ability of nanomaterials to enhance properties of polymers [63, 64]According to López de Dicastillo et al. [54], recyclability evaluation of PNCs requires understanding of the polymer type, nanomaterial type and its concentration, the morphology of the material, the presence of additives, and the conditions of thermal–mechanical cycles. Similarly, while providing value-added products from plastic waste nanocomposites, their recyclability should be taken into consideration [54].

According to Sánchez et al. [63], one solution for PNC recycling would be to develop industrial processes to remove nanoparticles from the polymer melt. In addition, the effects of recycling cycles on the quality and properties of PNCs should be explored. Sidi Salah et al. [65] studied the effect of aging and mechanical recycling on virgin PLA/nanoclay composite films’ electromagnetic performance, which was not significantly affected by recycling practice carried out using extrusion and compression molding. Zhang et al. [64] studied the effect of recycling on the mechanical, chemical, and rheological properties of a virgin PC/CNT nanocomposite using repeated injection molding and granulating for up to twenty cycles. The study found a decrease in the melt viscosity and overall mechanical properties but no significant change in chemical structure or elasticity was observed. Compared to neat polymers, plastic waste NC exhibited greater decrease in properties as a result of recycling practice. The study highlighted that greater importance should be paid on to the issue of PNC recycling.

To summarize, the use of plastic waste as a secondary raw material is the preferred method over incineration and landfilling with respect to sustainability and the environment. The use of plastic waste instead of virgin polymers provides also advantages from the environmental and economical perspectives as energy intensive production of petroleum-based virgin polymers is avoided. Moreover, the improved material properties through nanomaterial inclusion leads to weight reduction and material savings, which has a beneficial effect on the transportation and usage phases. Nevertheless, the production of most nanomaterials has high energy demand emitting GHGs, which should be considered when evaluating the sustainability of plastic waste nanocomposites. Nanoclays, graphene, and carbon black can be considered as environmentally friendly nanomaterials as they have relatively low environmental impact compared to most other nanomaterials. Plastic waste treatment methods might hinder the sustainability benefits of the waste material while increasing the price. Therefore, further research on plastic waste nanocomposites in respect to sustainability and economics should focus on the use of plastic waste without any preparation processes, and to consider the plastic waste NC recyclability aspects.

## Safety of plastic waste and virgin polymer nanocomposites

Only around 7% of government fundings and literature on nanotechnology is focusing on the human health and environmental implications, and few studies provide clear statements on nanomaterial safety [66]. On the whole, nanomaterials are considered safe when encapsulated in a polymer matrix [14]. Nevertheless, even when tightly bound with the matrix, nanomaterial release is possible during the lifecycle of a NC. The risk of nanomaterial release exists during the production, usage and disposal stages. Accidental fires create uncontrolled nanomaterial release risk during the usage [15]. Once released, nanomaterials from PNCs may end up in the human body via inhalation, ingestion, or cutaneous exposure, and they find their way into the environment via combustion or landfilling [15, 67, 68].

Only little research has been conducted on polymer nanocomposites where plastic waste is used as a matrix, and even less work has studied their safety during manufacturing, usage, and end of life procedures. Currently, only few studies consider the safety of plastic waste NCs, which of all focus on the nanomaterial migration in food packaging applications. Therefore, the safety evaluation in this paper is mainly with respect to virgin polymer nanocomposites.

In general, plastic waste might contain contamination of foreign materials such as metal, paper and wood [50]. In addition, the plastic waste can contain hazardous chemical elements because of the unknow usage history [50]. As observed by Turku et al. [50], plastic waste from constructions and households obtained from landfill, revealed elements of magnesium and bromine, which are signs of fire retardants. Along other elements, they also observed titanium, which is widely used as pigment in plastic products. This highlights the issue dealing with the unknow composition and elements of plastic waste streams. Therefore, in order to evaluate the safety of plastic waste NCs, the safety of both components, the plastic waste and the nanomaterial, must be considered separately including the evaluation of their synergistic effects on safety aspects.

### Food contact safety and nanomaterial migration of polymer nanocomposites

Nanomaterial migration from packaging to food creates concern about their safety in food packaging applications. Safety evaluation can be carried out with migration tests using liquid food simulants [54] and the overall migration limit is determined in EU regulations on food contact plastics (Commission Regulation (EU) No 10/2011). Generally, the limit is not exceeded with virgin polymer nanoclay composites [69, 70]. However, Nasiri et al. [71] emphasize that evaluation of nanomaterial migration from food packaging is not enough to determinate all the safety risks, and nanocomposite packaging safety should be considered in terms of all interactions between polymers, nanomaterials, compatibilizers, modifiers, additives, and food simulants.

Nanoclays are commonly used nanomaterials in virgin PNCs for food contact applications because their platelet structure provides a barrier effect. With plate-like nanomaterials, the type of morphology, intercalated or exfoliated, influences the migration mechanisms of the nanomaterials, and the exfoliated structure can result in higher nanoclay migration compared to an intercalated structure [68]. In addition, the contact solution can affect the polymer structure, possibly exacerbating migration of contaminants [68].

A nanoclay surfactant, for example, alkyl ammonium ions, improves compatibility with the polymer matrix but may lead to health risks in food contact applications [72]. The type of modifier and the concentration of nanoclay can affect the toxicity of the PNC in respect to human cell lines via oral pathways [73]. The study carried out by Houtman et al. [73] revealed that one clay with specific modifications showed cytotoxic and genotoxic effects, whereas the other two clays studied were safe with respect to toxicity evaluation. Similar observations were made in the studies of Maisanaba [69] and Bandyopadhyay and Ray [74], where it was noted that the toxicity profiles and safety for food contact applications of nanoclays in PNCs were dependent on the modifier used.

There are only a few studies focusing on the safety aspects of plastic waste nanocomposites, which of all are focused on food packaging applications. According to Velásquez et al. [75], global substance migration and metal traces migration to food simulants of polymer nanocomposite films made of PET waste and nanoclays were below the limits determined in the EU legislation (10/2011). According to the review carried out by López de Dicastillo et al. [54] on nanomaterial safety with recycled polymers for food packaging, only nanoparticles that were close to the packaging material’s surface had potential to migrate into food. Also, the stronger the attachment between the matrix and nanomaterials, the lower the potential for migration [54]. Similarly, in the reviews of Störmer et al. [76] and Bandyopadhyay and Ray [74] on virgin PNC safety, it was noted that mechanical impact towards food contact plastic is a risk for nanomaterial release due to weak binding on the surface; however, the probability of nanomaterial migration from PNC packaging to food is very low when nanomaterials are completely encapsulated in the polymer matrix.

The smaller the particle size the greater its toxicity due to the high surface to volume ratio increasing the possibility of nanomaterials interacting with biological molecules [68]. In the critical review of Störmer et al. [76] on the migration potential of nanoparticles in food contact plastics, it was concluded that nanomaterials with particle diameter less than 2 nm (nm) have a real potential to migrate from plastic to food, while particles of size being greater than 5 nm, the migration potential is small even with high nanomaterial loadings.

As well as particle size, nanomaterial type, surface chemistry, impurities, and shape influence the potential for nanomaterial release from the polymer matrix. For example, even though CNTs and graphene nanoplatelets are both carbon-based nanomaterials, they have different structures, which affects their degradation and toxicological properties, and graphene with layered structure is theoretically preferred over CNTs in terms of safety [15]. In addition, exposure to environmental conditions such as UV radiation, moisture and mechanical stress can enhance polymer matrix degradation and nanomaterial release [14]. Therefore, the type of nanoclay, the modifier to be used, the particle size, nanomaterial concentration, and the type of cell line have an effect on the toxicity profile of PNCs [73, 74]. Nevertheless, it must be noted that nanoclays have also a positive impact on food safety when used in plastic packaging due to their ability to reduce migration of toxic compounds such as UV absorbers and plasticizers from the plastic to food [54].

### Product safety and nanomaterial release during PNC incineration and degradation

PNC incineration creates safety concerns on the nanomaterial release to the environment. During thermal decomposition of PNCs, two solid byproducts are generated, released aerosol and residual ash [67]. Both professionals, such as employees of incineration facilities and fire fighters in the case of accidental fires, and consumers can be exposed to these byproducts. Therefore, the use of high efficiency filters and wet scrubbers in waste incineration plants is essential to prevent nanomaterial release to the environment and into human bodies [67, 77].

Sotiriou et al. [67] studied the effect of incineration and thermal decomposition on nanomaterial release of polymer nanocomposite byproducts at their end-of-life. No nanomaterials were observed from the released aerosol when using organic nanomaterials. However, when using inorganic iron oxide nanomaterial, a minimal amount of iron was observed in the released aerosol, indicating potential for nanomaterial release from PNCs during incineration.

In the study of Janhäll et al. [78], PC and HDPE reinforced with MWCNTs were incinerated in a pilot-scale fluidized bed unit to investigate the nanomaterial release during combustion at temperatures from 750 to 850 °C, which are values defined in the EU’s waste incineration directive (2000/76/EC). The study revealed that MWCNTs may not be fully destroyed during combustion and nanomaterials are more abundant in the flue gas with PC than HDPE, indicating that the polymer matrix has a significant effect on the survival of nanomaterials during the combustion. In addition, the incineration temperature affected CNT survival during the process as more nanomaterials were detected in the flue gas with lower temperature. However, no CNTs were identified from the bottom ash [78]. Kotsilkov et al. [15] also observed large amounts of graphene NPs and MWCNTs in residual ash after combustion at 500 °C and 850 °C, indicating unacceptable to high safety risks depending on the temperature. Similarly, Bouillard et al. [77] observed MWCNT release from an acrylonitrile butadiene styrene (ABS) matrix at gas phase during combustion even at relatively low temperature (400 °C).

Although landfilling is a common method for material disposal, it is the least preferred recycling method in the EU’s waste hierarchy. Kotsilkov et al. [15] studied the risk of carbon-based nanomaterials release from a PLA matrix during degradation and observed very high release risk due to degradation of the biodegradable polymer matrix.

To summarize, nanoclay migration potential from a polymer matrix is dependent on multiple factors including the type of modifier, the particle size and nanomaterial concentration, mechanical impact, environmental conditions and the type of cell line or food simulant. The nanomaterial release and migration potential from virgin polymer or plastic waste matrices do not exist while nanomaterials are fully embedded into the polymer matrix. However, mechanical impact towards product prepared from PNC can increase the nanomaterial release potential, creating safety concerns during the product’s usage phase. Nanomaterial release risk during incineration is dependent on the combustion temperature, the nanomaterial type, structure and concentration, and the type of polymer matrix, among other factors. During incineration, nanomaterials are released from the polymer matrix even at low processing temperatures. The risks to human health and the environment can be mitigated with efficient filters; however, nanomaterial release cannot be controlled during accidental fires. Landfilling is often used as a disposal method for PNC products but involves nanomaterial release risk especially when biodegradable polymers are used. Finally, plastic waste nanocomposite safety can be considered an under-researched area, and there is a need for further research on both virgin PNC and plastic waste NC safety, the synergistic effect of plastic waste and nanocomposite on safety aspects, and the development of national regulations and guidance [15, 71, 76, 78].

## Conclusion

Around 350 million metric tons of plastic waste is generated annually, and the amount is increasing every year, resulting in huge problems with waste stream management. The properties of plastic waste might deteriorate during mechanical recycling, limiting the applications of plastic waste as a secondary material. Adding nanomaterials into plastic waste using nanocomposite processing can enhance the properties of recycled polymers, enabling them to be used with a wider range of applications, and thus supporting sustainability, circular economy, and EU’s waste hierarchy.

Mechanical, thermal, electrical, shielding, and barrier properties, water absorption, and flame retardancy of plastic waste can be improved with low loadings of nanomaterials, of which nanoclays and MWCNTs are the most studied ones. The use of compatibilizers can improve the compatibility between the recycled polymer matrix and nanomaterials, hence improving nanomaterial dispersion, preventing agglomeration, and therefore improving the properties of plastic waste. Nevertheless, only relatively low nanomaterial loadings can be dispersed into virgin and waste-derived polymer matrices homogenously without agglomeration, which have a significant effect on the properties’ enhancement of the composite materials. Therefore, further research on nanomaterial dispersion methods and PNC processing are required.

The improved material properties of plastic waste allow their use in value-added applications for example in electronics, thermal management, and water treatment, and for filtration, shielding, and food packaging. However, these applications, which are proposed in the literature of plastic waste NCs, are not able to utilize the huge volumes of plastic waste produced annually. In addition, plastic waste utilized in most plastic waste NC papers do not reflect the realistic quality and composition of the solid plastic waste streams. Also, the pretreatment methods to purify plastic waste before nanocomposite processing increases costs and reduces sustainability gains. In addition, production of nanomaterials, especially CNTs, are expensive and energy intensive, reducing the environmental benefits gained from using plastic waste rather than virgin polymers.

In order to increase sustainability and improve the economics of plastic waste NCs, plastic waste should be utilized with minimum pretreatment steps, sustainable nanomaterials should be considered, and possible applications should be scouted to match the high plastic waste volumes, while keeping the costs competitive with virgin materials. Further research should additionally examine the health risks and environmental safety of plastic waste nanocomposites since potential exists for nanomaterial release during the PNC life cycle, and because of the unknow composition of the plastic waste, including the nanomaterials and plastic waste synergistic effect on safety aspects. Also, national regulations and guidance related to PNCs are needed. All in all, plastic waste nanocomposites have received relatively little study, indicating a need for further research in many areas from material selection to disposal strategies and everything in between.

## Data Availability

The authors declare that the data supporting the findings of this study are available within the paper.
